# Design and functional characterization of recombinant chimeras as vaccine candidates against *Lawsonia intracellularis*

**DOI:** 10.1007/s10529-026-03786-6

**Published:** 2026-09-18

**Authors:** Ana Vitória Costa, Neida L. Conrad, João Pedro G. Greco, Frederico S. Kremer, Fábio P. L. Leite

**Affiliations:** Programa de Pós-Graduação em Biotecnologia, Centro de Desenvolvimento Tecnológico, Universidade Federal de Pelotas (UFPel), Campus Capão do Leão, Prédio 19, Capão do Leão, Pelotas, RS 96010-900 Brasil

**Keywords:** Immunogenicity, Reverse vaccinology, Recombinant protein, Proliferative enteropathy

## Abstract

Proliferative enteropathy is a gastrointestinal disease caused by the intracellular bacterium *Lawsonia intracellularis*, affecting swine populations worldwide and leading to significant economic losses due to reduced growth performance, increased mortality, and treatment expenses. Current vaccines present limitations related to production processes, stability, and overall cost. This study aimed to design and evaluat chimeric proteins containing immunogenic epitopes of *L. intracellularis*. A recombinant chimera, designated Li_full, was rationally designed in silico by integrating predicted B-cell and MHC epitopes relevant to swine immunity. To further investigate the immunogenic potential of distinct regions, Li_full was subdivided into three fractions (Li_1, Li_2, and Li_3). All constructs were cloned into an *Escherichia coli* expression system, allowing efficient production and biochemical characterization of the recombinant proteins. Antigenicity was confirmed by strong reactivity with sera from naturally infected pigs. Immunogenicity was assessed through vaccination in mice, which developed significantly (p < 0.05) elevated antibody titers, demonstrating the ability of the chimeras to elicit robust humoral immune responses. The integration of bioinformatics-driven epitope prediction with recombinant DNA technology represents a promising and innovative strategy for controlling proliferative enteropathy. This study underscores the value of in silico design strategies for the development of targeted veterinary immunogens and provides a foundation for further research aimed at evaluating protective efficacy under field conditions.

**Supplementary Information:**

The online version contains supplementary material available at https://doi.org/10.1007/s10529-026-03786-6.

## Introduction

Proliferative enteropathy (PE) is a gastrointestinal disease that affects swine worldwide, causing substantial economic losses in production systems due to reduced weight gain, increased mortality, and the need for therapeutic interventions (Holtskmp 2020; Resende et al. 2015). Outbreaks of PE are estimated to result in losses of up to US$5 per infected animal, directly impacting the efficiency and profitability of the swine industry (Holtskmp 2020). PE is recognized as an emerging and persistent concern in modern intensive pig production systems, where high stocking densities and stress-related factors can facilitate disease outbreaks.

This disease manifests in two distinct clinical forms. The acute form is characterized by hemorrhagic diarrhea and rapid mortality, often leading to sudden losses within affected herds (McOrist et al. 1995; Vannucci and Gebhart 2014). The chronic or subclinical form is more insidious, being primarily associated with reduced growth performance in pigs and an increased incidence of stillbirths in pregnant sows (Guedes et al. 2017). The etiologic agent of PE is *Lawsonia intracellularis*, an obligate intracellular, Gram-negative, curved bacillus that possesses a single long unipolar flagellum, which confers motility upon release from infected enterocytes (Lawson and Gebhart 2000). Infection predominantly affects the ileum and colon of susceptible pigs, where the bacterium invades enterocytes and resides in the apical cytoplasm of intestinal crypt cells. This leads to pronounced epithelial hyperplasia, distortion of mucosal architecture, and subsequent impairment of intestinal function (McOrist et al. 1995). These pathological alterations reduce absorptive capacity and contribute to the clinical manifestations observed in both acute and chronic disease presentations.

Although antimicrobial therapy is available, sustainable long-term control of proliferative enteropathy relies primarily on vaccination. Currently, both live attenuated and inactivated vaccines are commercially available, Enterisol ileitis® (Boehringer-Ingelheim) and Porcilis Ileitis® (Intervet, Merck), respectively. However, the production of these vaccines is limited to a few laboratories due to the complexity of the culture systems required for *L. intracellularis* propagation, which involves suspension cell cultures maintained under specialized, gas-controlled conditions (Roerink et al. 2018; Vannucci et al. 2012). Furthermore, existing vaccines face limitations related to stability, efficacy, and scalability, highlighting the need for more accessible, cost-effective, and broadly applicable immunization strategies (Holyoake et al. 2009; Kruse et al. 2017; Riber et al. 2015).

A promising alternative to overcome these limitations is the development of recombinant vaccines based on *L. intracellularis* immunogenic proteins identified through reverse vaccinology approaches (Montesino et al. 2019; Soria-Guerra et al. 2015). Reverse vaccinology employs bioinformatic tools to screen the pathogen’s genome for candidate epitopes, enabling the rational selection of highly immunogenic antigens for vaccine design. This approach has been successfully applied to the development of vaccines against several pathogens, including *Neisseria meningitidis* and *Streptococcus pneumoniae*, and holds significant potential for advancing immunization strategies against *L. intracellularis* (He et al. 2010; Pizza et al. 2000; Seib et al. 2012). By eliminating the need for whole-cell bacterial cultivation, reverse vaccinology enables the creation of epitope-based vaccines that elicit robust and targeted immune responses while improving scalability, safety, and production efficiency.

Therefore, this study aimed to design, construct, and characterize chimeric proteins containing *L. intracellularis* epitopes and to evaluate their immunogenicity through integrated in silico, in vitro, and in vivo approaches.

## Materials and methods

### Data acquisition

Eighteen genomes of *Lawsonia intracellularis* were obtained in GenBank format from NCBI using the Datasets tool (https://www.ncbi.nlm.nih.gov/datasets/), with the following accession codes: GCA_000055945.1, GCA_000331715.1, GCA_001975945.1, GCA_003312265.1, GCA_003312285.1, GCA_003312305.1, GCA_008363085.1, GCA_020005205.1, GCA_025736895.1, GCF_000055945.1, GCF_000331715.1, GCF_001975945.1, GCF_003312265.1, GCF_003312285.1, GCF_003312305.1, GCF_008363085.1, GCF_020005205.1, and GCF_025736895.1.

### Pangenomic analysis

The sequences were converted to GFF3 format using the BioPerl package (https://bioperl.org/) and then processed by the Roary tool, executed with default settings, to identify orthologous groups among the different strains and then separate the genes belonging to the core genome (conserved in at least 95% of strains). From this analysis, fractions in the *L. intracellularis* pangenome were identified.

### Reverse vaccinology and immunoinformatics

Based on the core genome, the structural and functional annotation of proteins encoded by its representative sequence (as defined by Roary) was performed to identify signal peptides, transmembrane helices, β-barrel structures, and cellular localization using the SignalP, TMHMM, TMBED, and PSORT tools, respectively. The identification of swine B-cell and MHC-I epitopes was carried out using the EpiDope and NetMHC tools (with swine MHC models).

Proteins containing a signal peptide and predicted to be located in the outer membrane (identified by the absence of transmembrane helices, the presence of β-barrel structures, and subcellular localization prediction) represented five orthologous groups. From these, contiguous regions containing overlapping swine B-cell and MHC-I epitopes were extracted and merged into single antigenic selections.

The antigenic regions were then assembled into a single chimeric construct, referred to as Li_full (76.30 kDa), connected by glycine linkers (GGGGG). To determine whether individual portions of the chimera could independently trigger an immune response, Li_full was further divided into three derived fractions, designated Li_1, Li_2, and Li_3.

The tertiary structures of the chimera and its fractions were predicted using I-TASSER, based on their respective primary sequences (Yang et al. 2015; Yang and Zhang 2015). The physicochemical properties of the recombinant chimera were evaluated using the ProtParam tool, including GRAVY (Grand Average of Hydropathicity), molecular weight, instability index, half-life, aliphatic index, isoelectric point (pI), amino acid composition, and total number of positive and negative residues (Gasteiger et al. 2005). Additionally, hydrophobicity profiles were analyzed using the ProtScale server (Gasteiger et al. 2005).

### In silico* assembly of plasmids*

After identifying immunodominant epitopes and selecting the most promising ones for multi-epitope vaccine formulation, these were connected by glycine linkers containing five repetitions of this amino acid. The full chimeric construct (Li_full) and three constructs corresponding to its individual fragments (Li_1, Li_2, and Li_3) were synthesized by GenOne Biotechnologies (Rio de Janeiro, Brazil) and delivered cloned into the pET-28a(+) vector. All sequence data are provided in the Supplementary Material (Figure S1).

### Expression and purification of recombinant antigens

The synthetic plasmids containing the sequences of interest were introduced into competent *Escherichia coli* BL21 Star™ cells by electroporation. After transformation, recombinant cells were selected on Luria–Bertani (LB) (Sigma-Aldrich) agar plates supplemented with kanamycin (Sigma-Aldrich). Individual colonies were selected for pre-inoculation in 15 mL of liquid LB medium containing kanamycin (100 μg/mL) and incubated on a rotary shaker (120 rpm) for 12 h at 37 °C. The pre-inoculum was transferred to an Erlenmeyer flask containing 500 mL of LB medium supplemented with kanamycin (100 μg/mL) and incubated at 37 °C under agitation (150 rpm) until reaching an optical density (OD_600_) of 0.6–0.8. Protein expression was induced at the logarithmic growth phase by adding 1 mM IPTG (Sigma-Aldrich), followed by incubation for an additional 4 h at 37 °C.

After expression, bacterial cultures were centrifuged, and the cell pellets were resuspended in wash buffer (50 mM sodium phosphate, 300 mM NaCl, and 20 mM imidazole, pH 8.0) supplemented with lysozyme (1 mg/mL). The suspension was maintained under agitation for 1 h under the same conditions previously described. Subsequently, samples were incubated on ice for 20 min and subjected to sonication using cycles of 20 s ON and 15 s OFF. The lysate was centrifuged at 10,000g for 10 min, and the resulting pellet was resuspended in wash buffer containing 8 M urea and maintained under agitation overnight to promote protein solubilization. After an additional centrifugation step, the supernatant was filtered through a 0.22 μm membrane filter (Kasvi) and applied to a nickel affinity chromatography column (HisTrap™ HP, Cytiva) for purification of His-tagged recombinant proteins. Bound proteins were eluted using an imidazole-containing buffer according to the manufacturer’s recommendations. Following purification from inclusion bodies under denaturing conditions, the recombinant proteins were refolded by stepwise dialysis through decreasing concentrations of urea (6, 4, 2, 1, and 0 M) in PBS at 4 °C (Yamaguchi and Miyazaki 2014). After the final dialysis against PBS for 24 h, the proteins were stored at −80 °C or 4 °C until further use.

### Characterization of recombinant antigens

Protein characterization was performed using fractions obtained after nickel affinity purification. Recombinant protein purity was evaluated by 12% SDS-PAGE followed by Coomassie Brilliant Blue staining. For immunodetection, proteins separated by SDS-PAGE were transferred to a nitrocellulose membrane and blocked with 5% skim milk prepared in phosphate-buffered saline (PBS). Membranes were incubated with a monoclonal anti-histidine antibody (Anti-6xHis, Sigma-Aldrich) at a dilution of 1:6000, followed by incubation with a peroxidase-conjugated anti-mouse secondary antibody (Sigma-Aldrich) at a dilution of 1:10,000. Protein detection was achieved by developing the membrane with a DAB (diaminobenzidine) solution (0.6 mg of diaminobenzidine, 0.03% nickel sulfate, 50 mM Tris–HCl (pH 8.0), and 15 μl of hydrogen peroxide). Protein concentration was determined using the Bradford Protein Assay Kit (Sigma-Aldrich), according to the manufacturer’s protocol.

### Evaluation of recombinant constructions’ antigenicity

To evaluate the antigenic potential of the recombinant chimera and its fractions, the proteins were tested against sera from naturally infected pigs from pigs and mice vaccinated with the commercial vaccine Enterisol ileitis® (Boehringer-Ingelheim). Protein antigenicity was initially assessed by Dot blot analysis, in which 100 µg of each protein sample was applied onto a nitrocellulose membrane. The membrane was subsequently blocked with 5% (w/v) skim milk powder diluted in PBS. Sera from infected and vaccinated animals at a 1:50 dilution were incubated with the membrane, followed by incubation with anti-pig and anti-mouse immunoglobulins (Sigma-Aldrich) diluted 1:3000 and 1:5000, respectively. After washing, the reactions were visualized using a DAB solution (Sigma-Aldrich, 0.6 mg diaminobenzidine, 0.03% nickel sulfate, 50 mM Tris–HCl, pH 8.0, and 15 μL hydrogen peroxide).

### Formulations and administration of chimeras as experimental vaccines

The immunogenicity of the experimental vaccines was evaluated in a mouse (Balb/c) model. A total of 50 mice were divided into 5 experimental groups: Group 1. Control, receiving saline solution with 10% Al(OH)₃; Group 2. purified Li_1 protein; Group 3. Li_2; Group 4. Li_3; Group 5. Li_full. All groups, including the control, were administered a 200 µl dose subcutaneously. The experimental vaccines consisted of 100 µg of the respective recombinant protein formulated with 10% Al(OH)_3_, and each group received two doses with a 21-day interval between injections. Blood samples were collected five times via submandibular vein puncture at 10-day intervals, from day 0 to day 40, to obtain serum for the evaluation of the humoral immune response.

### Evaluation of humoral immune response

Humoral immune response against recombinant antigens was evaluated through an indirect ELISA, in which antigen concentrations and serum dilutions were determined in checkerboard assays. Plates were coated with 100 ng/well of the respective recombinant proteins (Li_1, Li_2, Li_3, and Li_full) diluted in 0.1 M carbonate-bicarbonate buffer (pH 8.0) and incubated overnight at 4 °C. Serum was diluted 1:100 in PBS and applied to the plates in triplicate. Subsequently, peroxidase-conjugated anti-mouse IgG antibodies (Sigma-Aldrich), diluted 1:10,000 in PBS, were added. For detection, 100 µL of substrate solution (10 mL of 0.05 M phosphate–citrate buffer, pH 5.0, containing 0.004 g o-phenylenediamine and 15 µL hydrogen peroxide) was added to each well, and the reaction was allowed to proceed for 15 min in the dark at room temperature. The reaction was stopped by adding 50 µL of 2N H_2_SO_4_ per well. Absorbance was measured at 492 nm using an EZ Read 400 microplate reader (Biochrom, UK).

Antibody titration against the recombinant Li_full protein was also evaluated through an indirect ELISA. Plates were coated with 100 ng/well of Li_full diluted in carbonate–bicarbonate buffer (pH 8.0) and incubated for 18 h at 4 °C. Serum samples were tested in serial dilutions (1:50, 1:100, 1:200, 1:400, 1:800, 1:1600, and 1:3200) in duplicate. The remaining steps were performed as described above.

Data were analyzed using GraphPad Prism 8 software (GraphPad Software, CA, USA). Statistical analysis was performed using two-way ANOVA followed by Tukey’s multiple comparison test, considering p < 0.05 as statistically significant.

### Ethical aspects

All animal experiments were conducted following the guidelines of the National Council for Animal Experimentation Control (CONCEA). This study was approved by the Ethics Committee on Animal Experimentation of the Federal University of Pelotas (CEUA 007283/2023-73).

## Results

### In silico* characteristics*

Converted genomic sequences were identified in orthologous groups among different bacterial strains. Table 1 provides a detailed analysis of the fractions in the *L. intracellularis* pangenome, classifying orthologous groups based on their occurrence in different strains. The number of orthologs present in the core genome was 1202, representing highly conserved and essential genes found in all analyzed strains (95% or more). The soft-core genome had 112 genes, while the shell fraction had 140 orthologs. The cloud fraction of the analysis presented 60 orthologous genes.

**Table 1:** Pangenome composition of *L. intracellularis* based on orthologous gene clustering

Fraction	Number of orthologs	Strain occurrence (%)
Core genome (strict)	1202	95–100
Core genome (soft)	112	94–95
Shell genome	140	15–95
Cloud genome	60	0–15
Pangenome	1514	0–100

Core genome (strict) includes genes present in all strains; soft core includes genes present in nearly all strains

In the pangenome, 1514 orthologous genes were identified occurring in at least 1 of the *L. intracellularis* strains. The sum of all categories above includes all orthologous groups identified in all analyzed strains. This comprehensive representation of the pangenome covers the total diversity of genes found in different *L. intracellularis* strains.

### Genomic analysis

The selected protein sequences were characterized based on their structural and localization features using multiple bioinformatic tools. Analysis with the SignalP server revealed that three out of the five proteins possess a LIPO (Sec/SPII) signal peptide, suggesting that these proteins are directed to the secretion pathway and undergo specific lipoprotein processing during transport. In contrast, the remaining two proteins contain a SP (Sec/SPI) signal peptide, indicating secretion without subsequent lipoprotein modification. This variation in signal peptide types suggests that the selected proteins may follow distinct secretion routes and post-translational processing mechanisms.

The prediction of transmembrane regions using TMHMM showed results ranging from “SP” to “BETA,” consistent with differences in secondary structure and the presence or absence of transmembrane helices or β-barrel motifs. According to TMBED analysis, all proteins were classified as outer membrane proteins (OMP), supporting their localization at the bacterial surface. Likewise, PSORT prediction confirmed an Outer Membrane localization for all proteins, reinforcing their potential biological relevance in surface exposure and host–pathogen interactions.

### Epitope prediction

The identification of B-cell and MHC-I epitopes was performed using EpiDope and NetMHC, the latter employing swine-specific MHC models. The predicted binding scores indicated variability among the analyzed proteins, suggesting the presence of distinct numbers and affinities of potential epitopes. When considering all alleles, the results pointed to the ability of the selected peptides to interact with multiple MHC-I molecules, supporting their relevance for immune recognition. Similarly, the number of predicted B-cell epitopes varied among the proteins, indicating that these regions are likely to be recognized by the immune system as potential targets for antibody responses.

The five orthologous groups identified represent distinct but evolutionarily conserved protein variants across species, reinforcing the potential functional conservation of these antigenic sequences. From these proteins, contiguous regions containing overlapping B-cell and MHC-I epitopes were extracted and merged into continuous antigenic regions. This approach resulted in 34 distinct regions, each containing at least one epitope predicted to elicit humoral or cellular immune responses.

These regions were subsequently assembled into a single chimeric construct, designated Li_full (76.30 kDa), in which the selected antigenic regions were connected by flexible glycine linkers (GGGGG). To assess whether smaller portions of the chimera could independently induce an immune response, Li_full was subdivided into three fractions representing different combinations of the selected epitope regions. The use of glycine linkers was intended to preserve conformational flexibility, while the generation of multiple constructs allowed the evaluation of different antigenic compositions for potential immunogenicity.

### Physicochemical parameters of the constructions

**Fig. 1:**
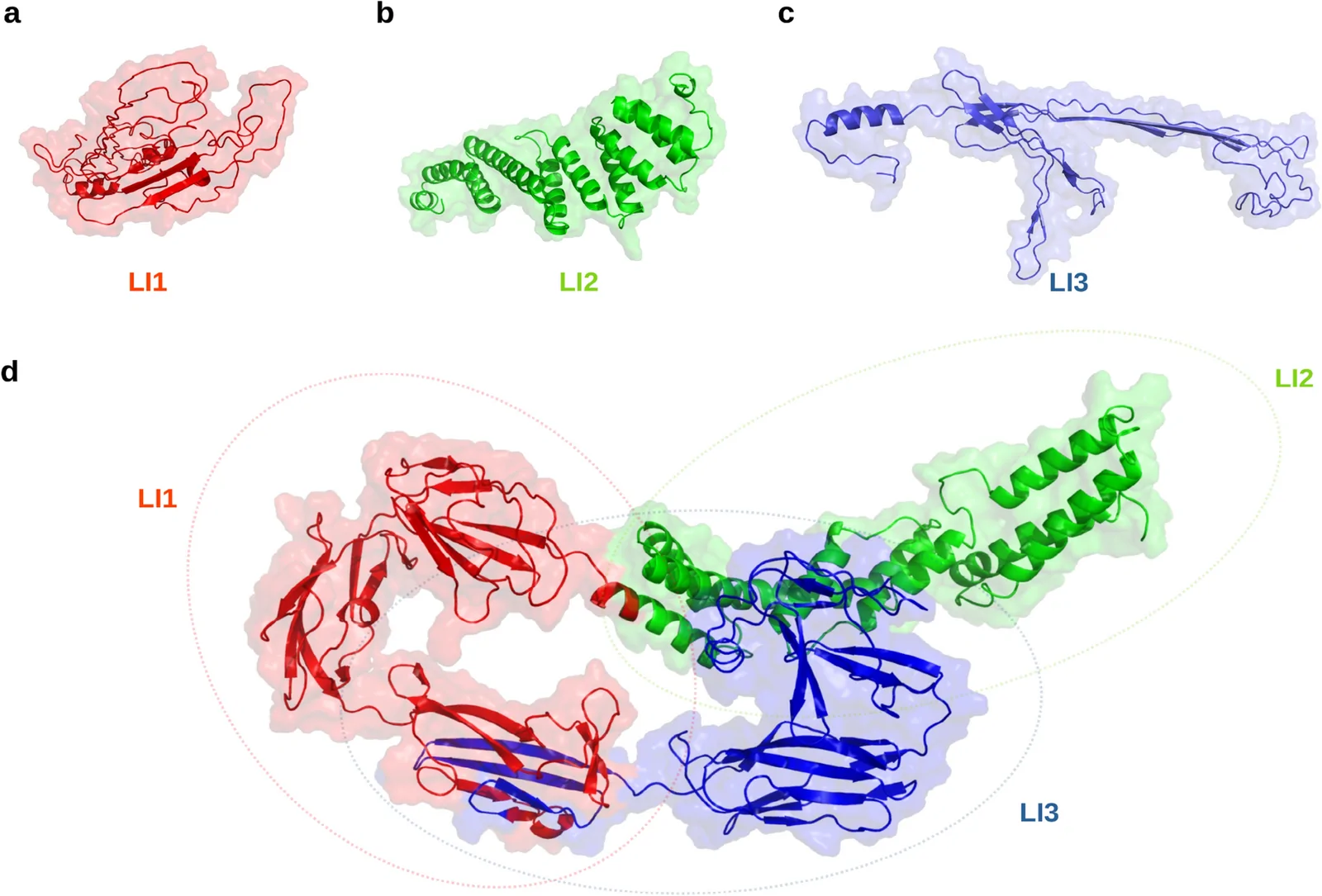
Tertiary structure prediction of the chimeric proteins using I-TASSER. **a** Li_1, **b** Li_2, **c** Li_3, and **d** Li_full

To assess the structural conformation of the recombinant proteins, the prediction of their tertiary structure was performed using I-TASSER, as illustrated in Fig. 1. The predicted models were analyzed based on their confidence scores (C-score) and structural alignment, providing insights into the stability and potential functional domains of the proteins.

Based on the analysis performed by ProtParam (Table 2), the molecular mass of the recombinant chimeras was evaluated. The pI values of the proteins ranged from 5.02 to 8.55. The estimated half-life of the proteins was over 10 h in the cytosol of *E. coli*. The half-life, which considers the total time required for a protein to disappear after being synthesized in the cell, was estimated to be 30 h for mammalian reticulocytes, > 20 h for yeast, and > 10 h for *E. coli*. The instability index, 45.94 (Li_1), 31.95 (Li_2), 50.18 (Li_3), and 42.45 (Li_full), indicated that only Li_2 would be stable (values below 40 indicate stability, while values above 40 indicate instability). This index provides an estimate of protein stability in a test tube, which can be a determining factor for effective production in heterologous expression systems (< or> 40). The best thermostability was 68.80 (Li_2), followed by 60.03 (Li_full), 57.76 (Li_3), and 57.71 (Li_1). In addition to being an indicator of thermostability, the aliphatic index is indicative of solubility in a cell when the protein is overexpressed.

**Table 2:** Physicochemical properties of chimeric proteins derived from *L. intracellularis*

Parameters	Li_1	Li_2	Li_3	Li_full
Amino acid length (aa)	210	255	223	696
Molecular mass (kDa)	24.2	27.7	24.0	76.3
Isoelectric point (pI)	5.34	5.02	8.55	5.70
Negative residues (Asp+Glu)	29	29	22	80
Positive residues (Arg+Lys)	23	24	24	71
Half-life (h) (*Escherichia coli*)	> 10 h	> 10 h	> 10 h	> 10 h
Instability index	45.94	31.95	50.18	42.45
Aliphatic index	57.71	68.80	57.76	60.03
Grand average of hydropathicity (GRAVY)	− 0.744	− 0.317	− 0.581	− 0.538

**Fig. 2:**
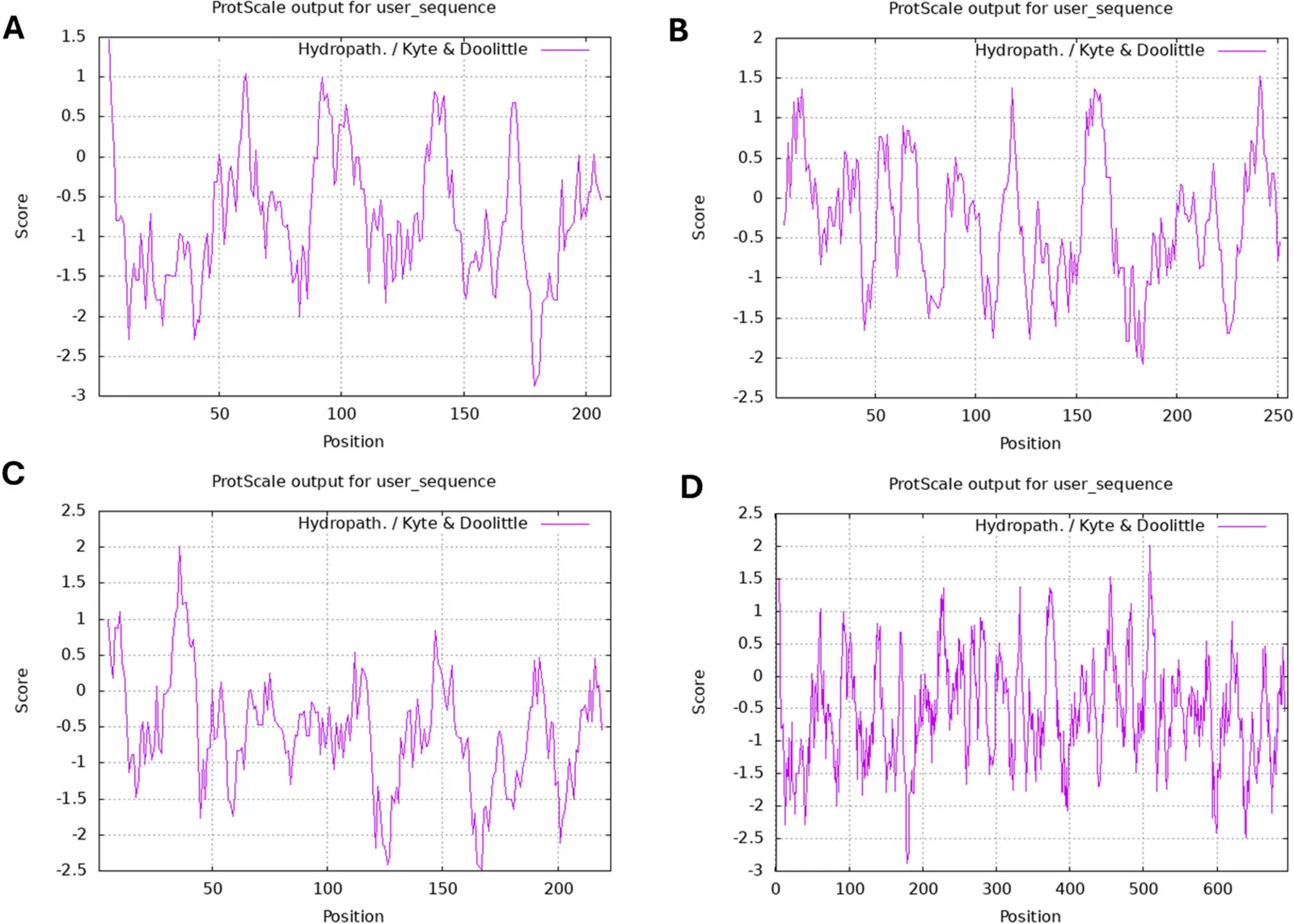
Hydrophobicity patterns of the recombinant chimeras. **A** Li_1, **B** Li_2, **C** Li_3 e, **D** Li_full

When analyzed by ProtParam (Table 2), the GRAVY index showed negative values, indicating hydrosoluble molecules. It is known that hydrophobicity is a type of interaction with a significant influence on protein folding, highlighting the need to quantify the side chains of amino acids by indices that show the level of hydrophobicity of each one. Thus, many researchers have been involved in creating ways to classify amino acids, leading to the development of hydrophobicity scales. For a better understanding of solubility, Fig. 2 presents the hydrophobicity plot of the proteins, where a predominant presence of hydrophobic regions can be clearly observed for Li_1, Li_2, and Li_full (positive regions), while Li_3 appears hydrophilic (negative regions).

### Expression and characterization of proteins

**Fig. 3:**
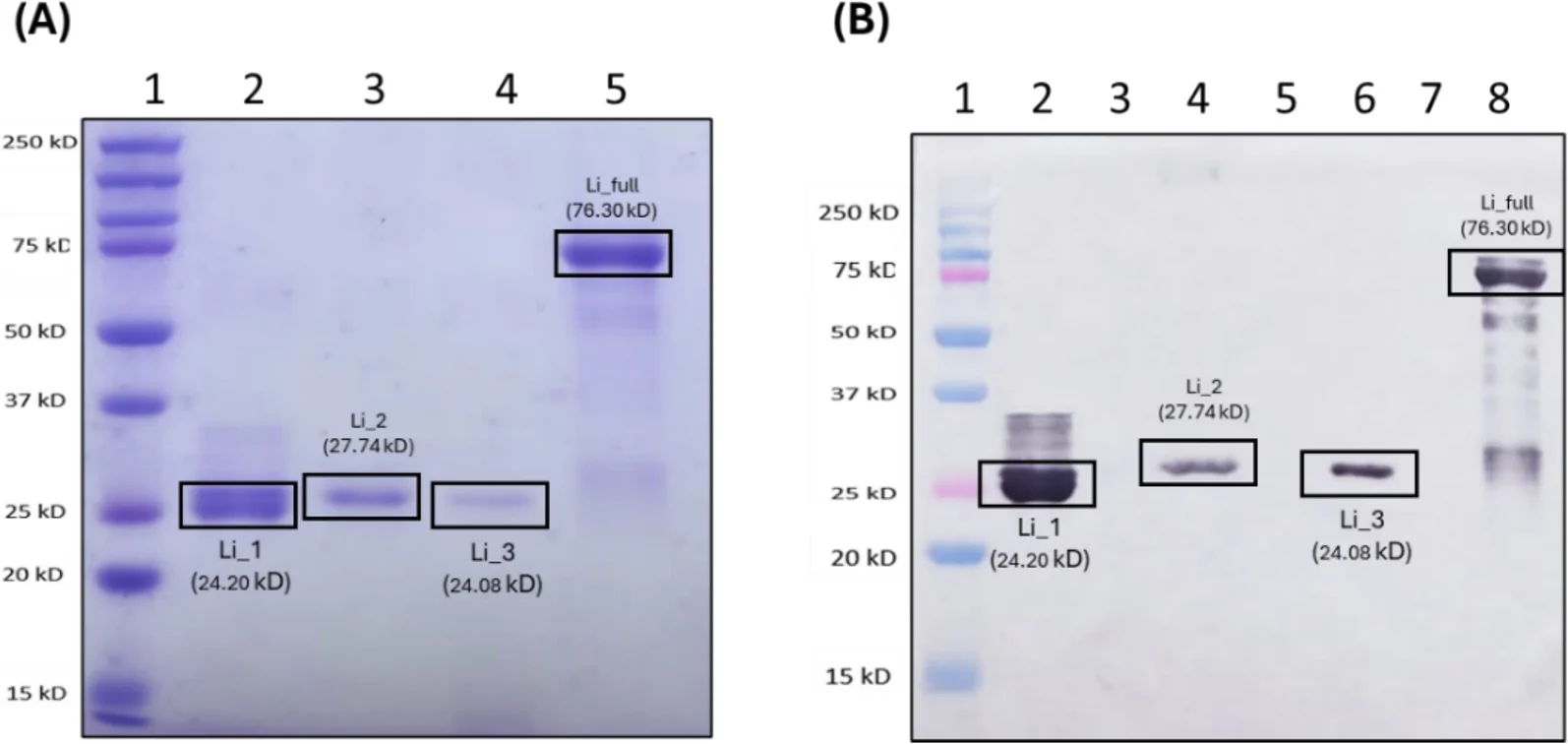
Expression and purification of recombinant Li proteins. **A** 12% SDS-PAGE of purified proteins: 1. Molecular weight marker (Bio-Rad); lanes 2–5 correspond to Li_1, Li_2, Li_3, and Li_full, respectively. **B** Western blot (15% polyacrylamide gel) using anti-His antibody: 1. Molecular weight marker (Bio-Rad); lanes 2, 4, 6, and 8 correspond to Li_1, Li_2, Li_3, and Li_full, respectively. Source: Author’s elaboration (2026)

Following the in silico analysis, the Li_1, Li_2, Li_3, and Li_full constructs were successfully cloned into synthetic plasmids and expressed in *Escherichia coli* BL21 Star cells, with recombinant proteins predominantly accumulating in the insoluble fraction. Distinct bands corresponding to approximately 24.20, 27.74, 24.08, and 76.30 kDa were consistently detected, in agreement with the predicted molecular masses of Li_1, Li_2, Li_3, and Li_full, respectively. The clear detection of these bands in the eluted fractions confirms the successful purification and integrity of the recombinant proteins (Fig. 3A and B).

Once the protein was solubilized using an 8 M urea buffer, slow dialysis with gradual reduction of solubilizing agent concentration was necessary. After dialysis, the protein concentration was determined by the Bradford Method. The expression yield (mg/L) of each protein is provided in Table 3.

**Table 3:** Expression yield of recombinant proteins

Recombinant protein	Expression yield (mg/L)
Li_1	15
Li_2	6
Li_3	10
Li_full	18

Values are approximate

### Antigenicity of recombinant proteins

**Fig. 4:**
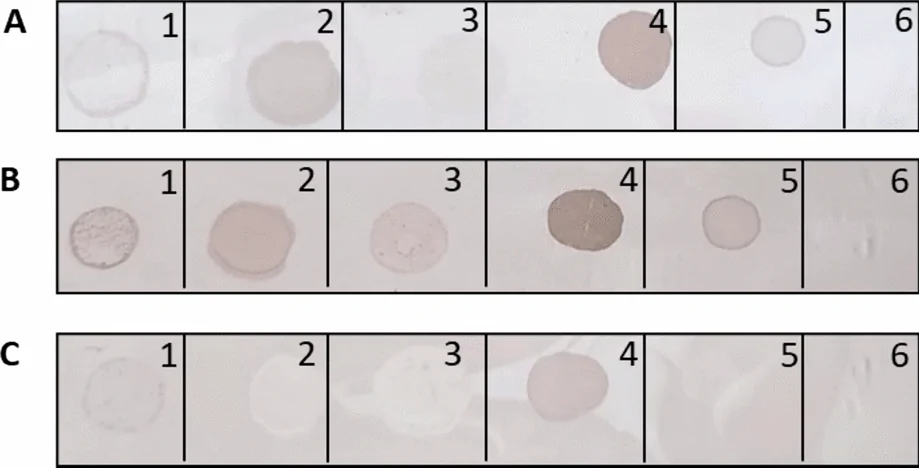
Antigenic recognition of recombinant proteins. Dot blot analysis showing recognition of recombinant antigens by sera from different sources: **A** naturally infected pigs; **B** pigs vaccinated with the commercial vaccine Enterisol ileitis®; C, mice vaccinated with Enterisol ileitis®. Spots correspond to: 1 – Li_1; 2 – Li_2; 3 – Li_3; 4 – Li_full; 5 – rLiTT; 6 – PBS (negative control)

All proteins were recognized by sera from pigs vaccinated with Enterisol ileitis® (Fig. 4), demonstrating that these constructed antigens may have vaccine potential in the species. Recombinant proteins Li_1, Li_2, and Li_full were also recognized by sera from naturally infected pigs (Fig. 4). Regarding sera from mice vaccinated with Enterisol ileitis®, only Li_full was capable of being recognized (Fig. 4). Additionally, a recombinant protein from *L. intracellularis* (rLiTT), constructed and tested previously by our group (Conrad et al. 2026, 2025), was added to be validated as a positive control, which was recognized by all groups. PBS was used as a negative control, which showed no reaction.

### Humoral immune response

**Fig. 5:**
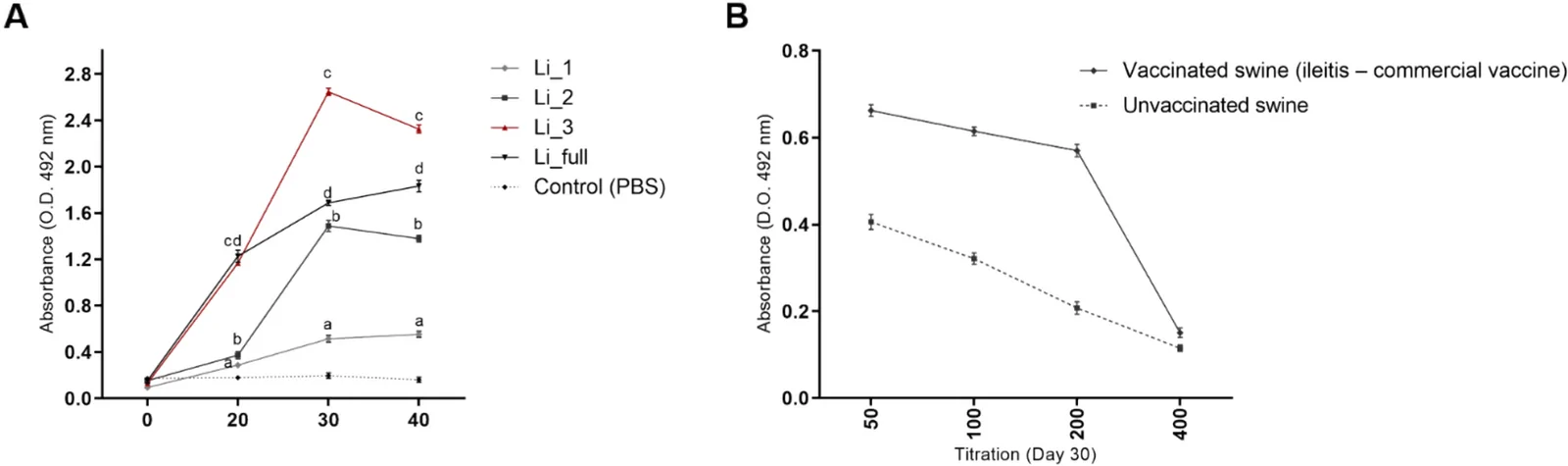
Dynamics of total IgG antibodies against the chimeric recombinant proteins. (A) ELISA detection of total IgG antibodies against Li_1, Li_2, Li_3, Li_full and Control (PBS). Animals were vaccinated on experimental days 0 and 21. Differences between groups were analyzed by two-way ANOVA followed by Tukey’s test (p < 0.05). Letters a, b, c, and d indicate statistical differences between vaccinated groups. (B) Antibody titration against Li_full using sera collected on experimental day 30, showing higher absorbance values in vaccinated pigs compared with non-vaccinated pigs. Data are presented as mean ± standard deviation (SD)

The immune response of vaccinated mice was evaluated using sera collected on days 0, 20, 30, and 40. The results indicated the immunogenicity of the recombinant proteins, showing the production of anti-rLi_1, anti-rLi_2, anti-rLi_3, anti-rLi_full IgG antibodies and Control (PBS) (Fig. 5A) after immunization. After the administration of a single vaccine dose on day 20 of the experiment, a 12-fold increase in antibody levels compared to day 0 was observed in the Li_3 and Li_full groups. With the second dose on day 30 of the experiment, a significant increase in antibody levels was observed, reaching a 27-fold increase in the Li_3 group and a 16-fold increase in the Li_full group. Despite the decrease observed on day 40, the Li_3 group still maintained a significant response. The Li_full group showed an increase in antibody levels 10 days after the second dose, with a 4-fold increase compared to day 30. Although the Li_1 and Li_2 groups demonstrated a lower response than the others, they showed a 4-fold increase compared to day 0. After the second dose, there was a gradual increase in absorbance observed in the Li_1 group. On the other hand, the Li_2 group showed a significant increase in antibody levels after the second dose, with a 14-fold increase on day 30 of the experiment, which remained significant until day 40 (p < 0.05). As expected, the PBS group did not develop detectable antibody titers, maintaining baseline levels throughout the experimental period. Consistent with the IgG responses observed for the chimeric proteins, the antibody titration against Li_full showed higher ELISA absorbance values in sera from vaccinated pigs compared with non-vaccinated pigs across the evaluated serum dilutions (Fig. 5B).

## Discussion

Advances in immunoinformatics have provided computational frameworks for antigen discovery and rational vaccine design (Soria-Guerra et al. 2015). Computational prediction of immunogenic epitopes, combined with pangenome analysis, enables the identification of conserved antigenic regions across bacterial populations while accounting for genomic variability that may influence pathogenicity and host adaptation (Medini et al. 2008). In this context, pangenome analysis of *Lawsonia intracellularis* highlights conserved and variable genetic elements that may contribute to bacterial adaptation, evolution, and pathogenicity (Cooper and Gebhart 1998). The presence of multiple predicted epitopes supports the immunogenic potential of these proteins.

All proteins analyzed in this study were predicted to be localized to the outer membrane, consistent with their proposed roles in secretion. Most proteins contain LIPO-type signal peptides, indicating targeting secretion pathways with potential lipoprotein processing, while others present SP-type signal peptides, suggesting secretion via the general pathway. Each protein also contains at least one predicted peptide capable of binding to swine MHC-I, as well as multiple B-cell epitopes. The diversity in epitope content further supports their immunogenic potential and suggests relevance in host–pathogen interactions (Hammer et al. 2020; Jespersen et al. 2017; Nielsen et al. 2003).

Outer membrane proteins are considered suitable vaccine targets because they are exposed to the host immune system and often participate in host–pathogen interactions, increasing the likelihood of antibody recognition and immune-mediated bacterial clearance (Aves et al. 2025; Li et al. 2025). As immunity against intracellular pathogens relies on CD8⁺ cytotoxic T lymphocytes capable of eliminating infected cells (Schmidt and Varga 2018), the chimera was designed by selecting contiguous regions containing overlapping B-cell and swine MHC-I epitopes. The induction of memory cytotoxic T cells is important for protection against intracellular pathogens (Enamorado et al. 2017; Huster et al. 2006; Klebanoff et al. 2006; Wherry and Ahmed 2004), and the inclusion of B-cell epitopes aimed to promote antibody production, which may contribute to bacterial neutralization and opsonization, facilitating pathogen clearance. In addition, the use of extended antigenic regions, rather than short isolated peptides, may facilitate antigen processing and presentation, potentially enhancing immune recognition across different SLA alleles in swine populations (Hammer et al. 2020; Skwarczynski and Toth 2016). In contrast to synthetic multi-epitope constructs composed of short peptides linked by artificial spacers, the present chimera was constructed from naturally contiguous antigenic regions, which may better preserve structural context and support antigen processing.

Based on these considerations, the design strategy prioritized the selection of antigenic regions combined into a single recombinant chimera (Li_full). To further assess the contribution of different antigenic segments, the construction was divided into three fractions (Li_1, Li_2, and Li_3), which were individually expressed, purified, and characterized. After confirming successful expression and purification, the antigenic properties of the recombinant proteins were evaluated.

Recognition of the recombinant chimera by sera from naturally infected pigs and from animals vaccinated with the Enterisol ileitis® vaccine indicates that the selected epitopes are likely expressed during natural infection and are immunologically relevant. Consistent with these findings, the Li_full chimera showed antibody recognition in ELISA assays, with titrated sera from vaccinated pigs presenting higher absorbance values compared to non-vaccinated controls. In addition, immunization of mice with Li_full induced IgG responses, with antibody levels maintained or increased after the second dose, indicating induction and boosting of the humoral response.

Although this study did not include an experimental challenge model, the observed pattern is consistent with previous reports associating IgG titers with protection against proliferative enteropathy (Fourie et al. 2021; Li et al. 2021; Montesino et al. 2019; Nogueira et al. 2013; Park et al. 2019), supporting the potential of these chimeras as vaccine candidates. The absence of a challenging study represents a limitation of the present work and was primarily due to the technical difficulties associated with the cultivation of *L. intracellularis*, an obligate intracellular bacterium that requires specialized cell culture systems for propagation. Furthermore, immunization with Li_full and its fractions induced antigen-specific antibody responses, demonstrating their ability to elicit humoral immunity. The antibody responses and recognition by sera from infected animals further support the relevance of the selected epitopes.

Previous immunoproteomic studies have identified *L. intracellularis* epitopes capable of inducing neutralizing antibodies, and multivalent recombinant vaccines have been shown to induce IgG responses and reduce histopathological lesions in experimental models (Fourie et al. 2021; Montesino et al. 2019; Obradovic et al. 2019). These findings warrant further assessment of the neutralizing capacity of antibodies induced by Li_full and its fractions. Overall, the results support the use of reverse vaccinology approaches and indicate that recombinant Li_full and its fractions can induce antigen-specific humoral responses. These findings provide a basis for future evaluation of protective efficacy under experimental challenge and field conditions.

Overall, this study highlights the potential of epitope-based vaccines guided by immunoinformatics as a strategy for the control of *Lawsonia intracellularis* infection.

## Conclusion

The use of bioinformatics tools enabled the construction of the chimera (Li_full) and its fractions as recombinant antigens against *L. intracellularis*. These proteins were efficiently expressed in *E. coli* BL21 Star™, and all demonstrated antigenicity. Immunization of mice induced a detectable humoral immune response, as evidenced by the presence of specific IgG antibodies. These results suggest that this approach may contribute to the development of promising vaccine candidates for pigs.

## Supplementary Information

Below is the link to the electronic supplementary material.

Supplementary file1 (PDF 668 KB)

## Data Availability

No datasets were generated or analysed during the current study.

## Declarations

### Supplementary material

Schematic representation of pET-28a(+) plasmid constructs encoding chimeric proteins derived from Lawsonia intracellularis. (A) Construct carrying chimera 1 (Li_1). (B) Construct carrying chimera 2 (Li_2). (C) Construct carrying chimera 3 (Li_3). (D) Construct carrying the full-length chimera (Li_full). Numbers indicate nucleotide positions. Key elements, including the T7 promoter, His-tag, and restriction enzyme sites, are indicated.
