# Supplementary material for: Design and functional characterization of recombinant chimeras as vaccine candidates against Lawsonia intracellularis
Source: Biotechnol Lett. 2026 Sep 18;48(5):115. doi: 10.1007/s10529-026-03786-6 (PMC13588837; doi:10.1007/s10529-026-03786-6)

Construct Map:

(A)

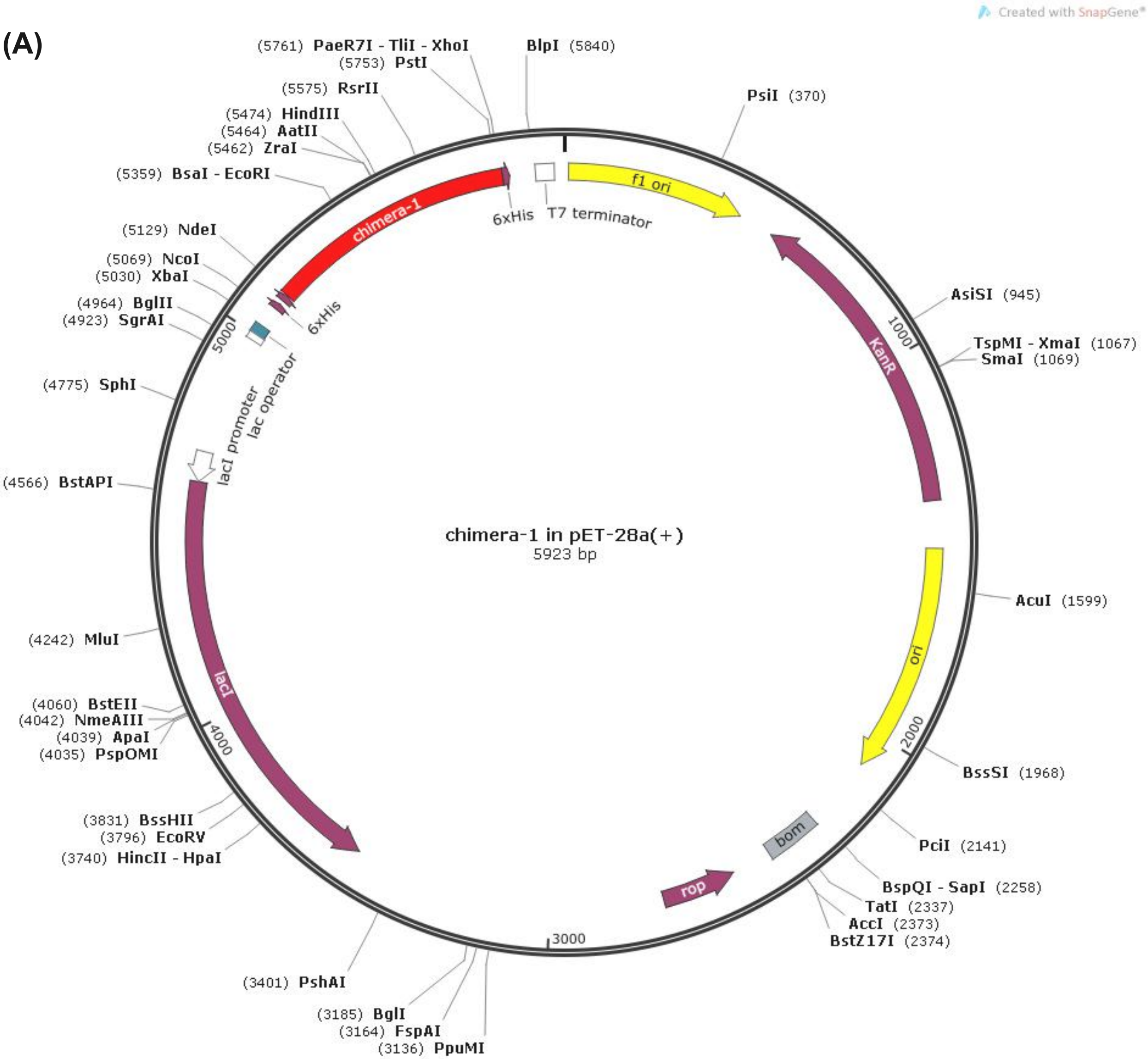

Construct Map:

(B)

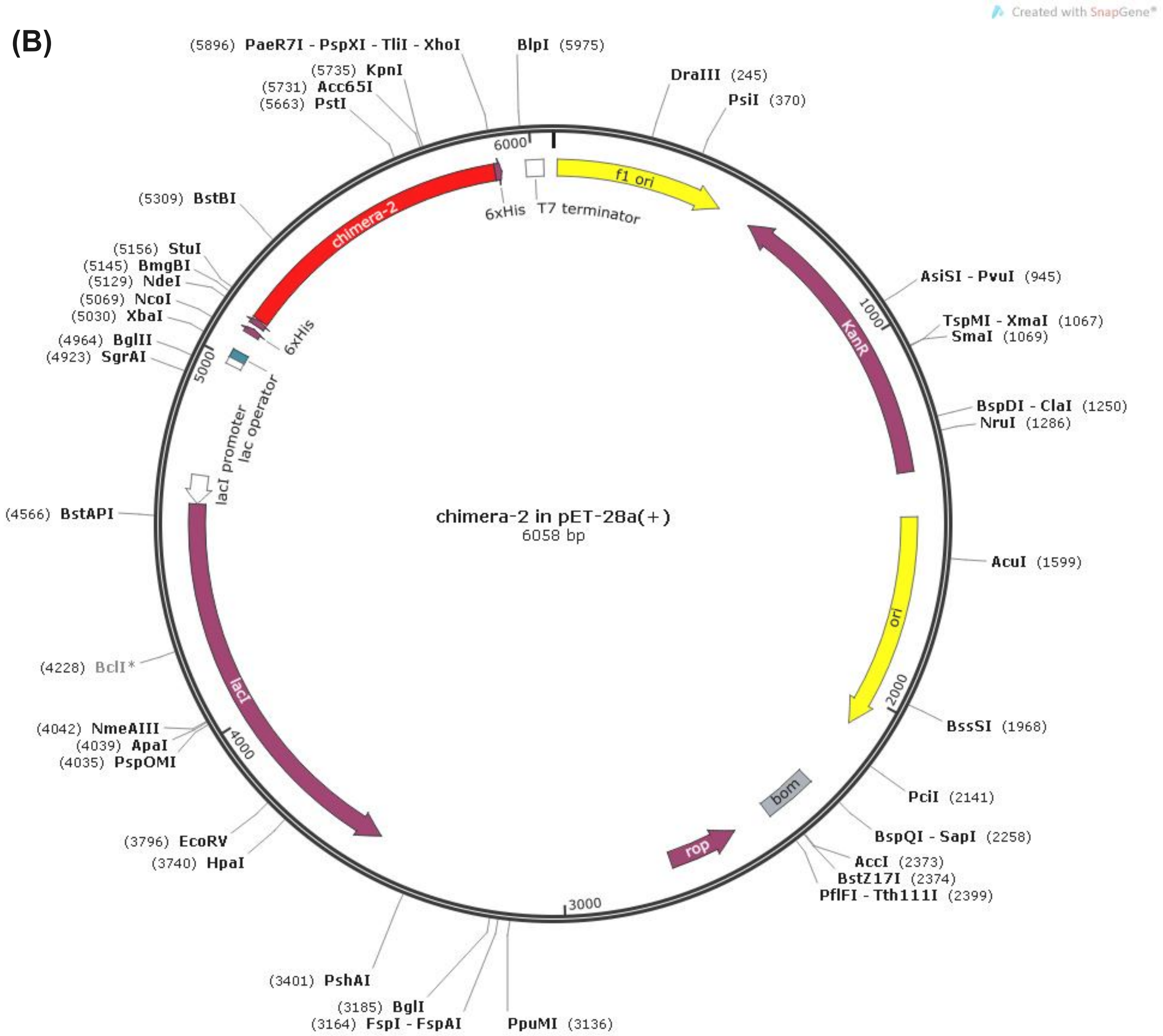

Construct Map:

(C)

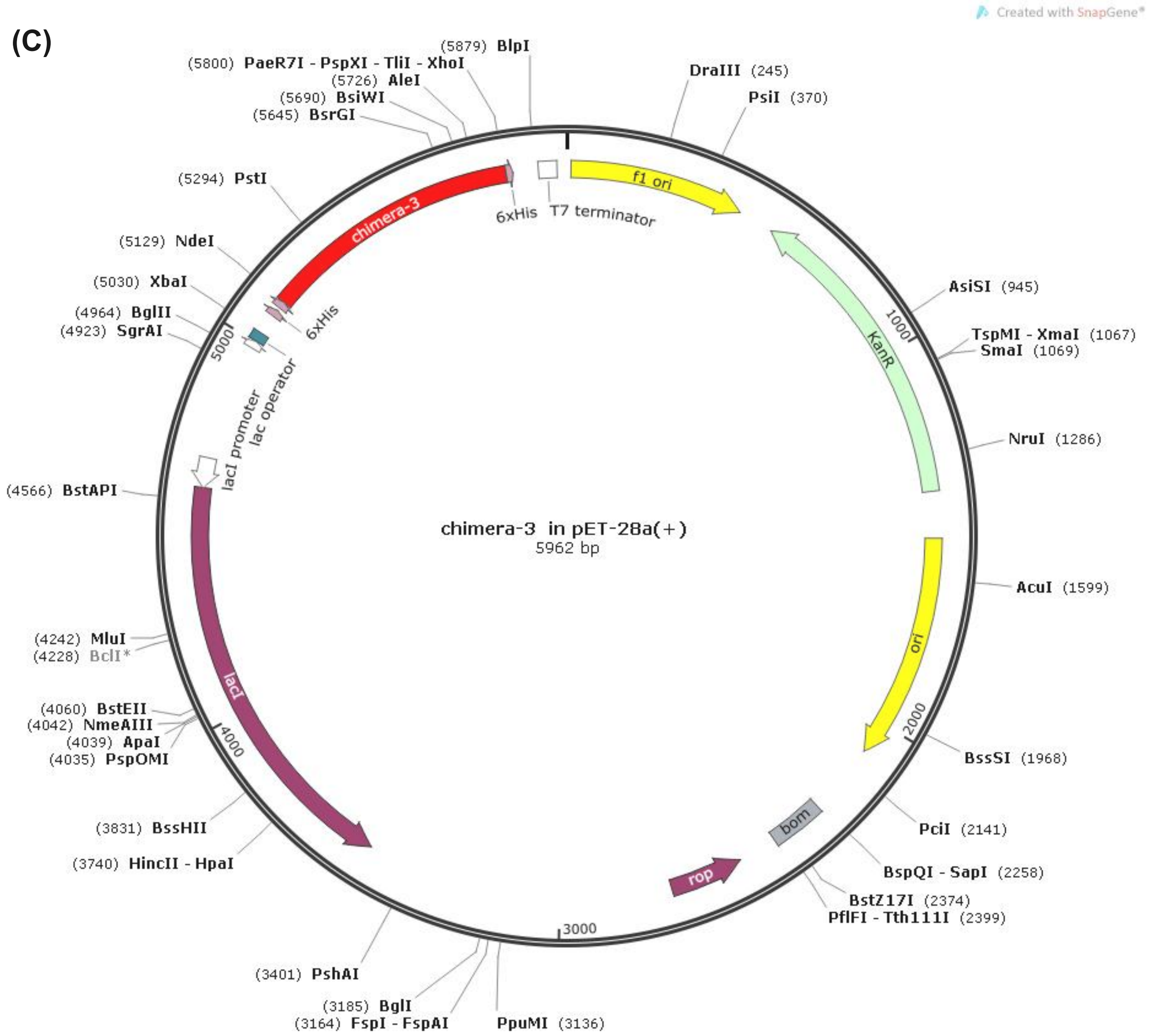

Construct Map:

(D)

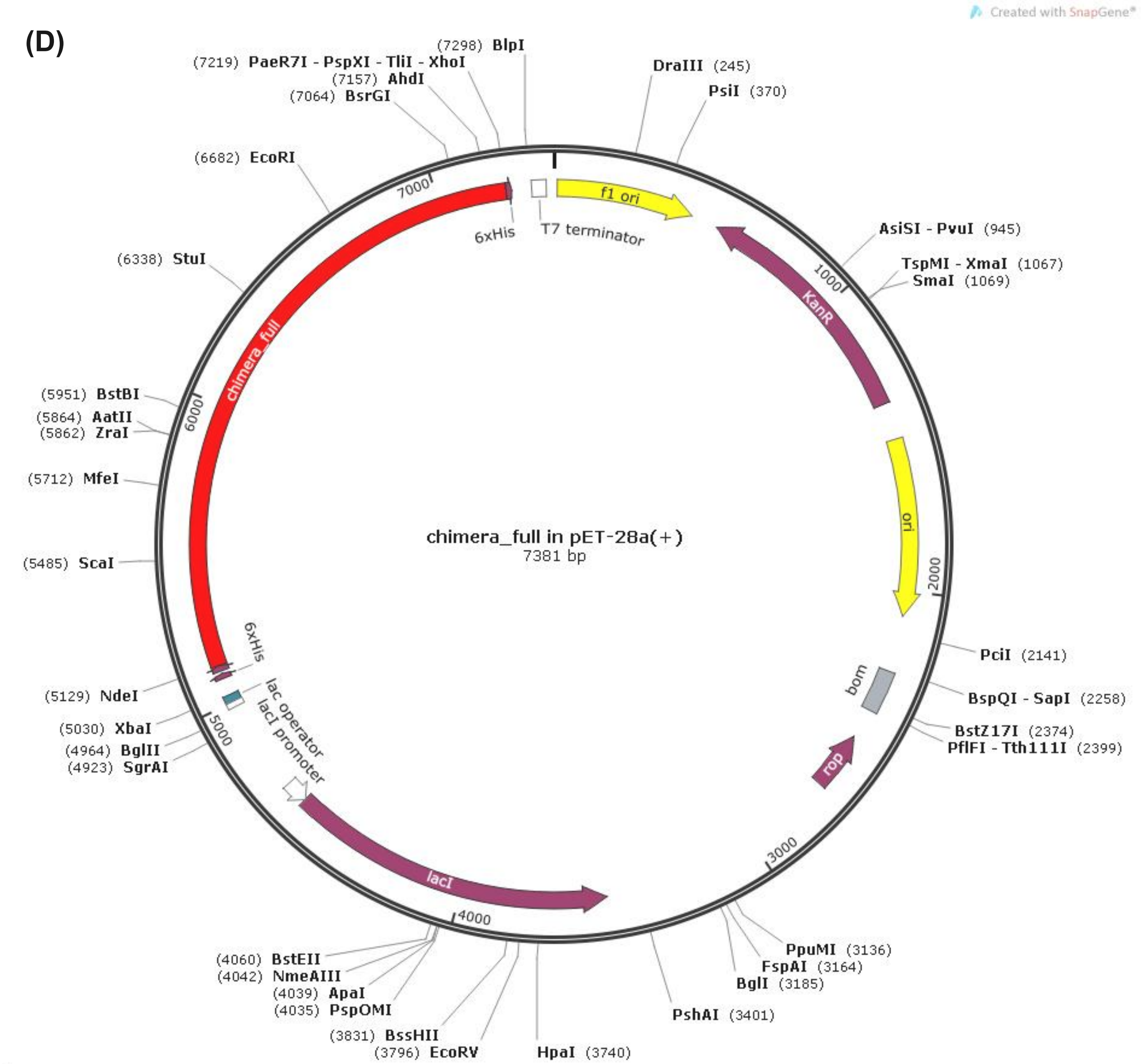

Supplement: Supplementary file 1 [file 10529_2026_3786_MOESM1_ESM.pdf]
